# Supplementary material for: Comprehensive Annotation and Functional Exploration of MicroRNAs in Lettuce
Source: Front Plant Sci. 2021 Dec 24;12:781836. doi: 10.3389/fpls.2021.781836 (PMC8739914; doi:10.3389/fpls.2021.781836)
Supplement: Supplementary file 2 [file Data_Sheet_8.ZIP › GSM2230749.plot/Lsa-miR171a_Lsat_1_v5_gn_3_681.1_713_TPlot.pdf]

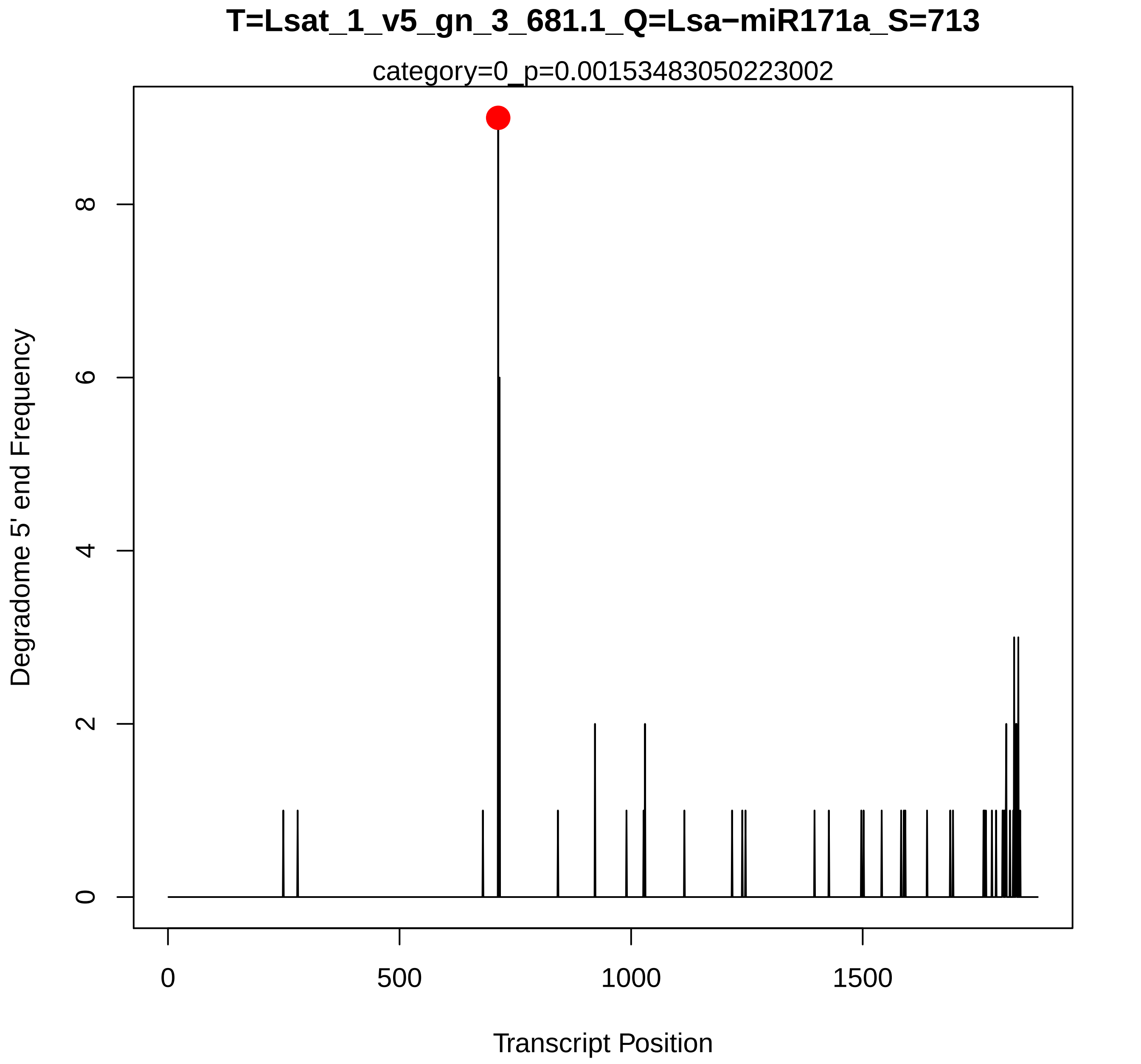
T=Lsat_1_v5_gn_3_681.1_Q=Lsa−miR171a_S=713
category=0_p=0.00153483050223002
Degradome 5' end Frequency
0
2
4
6
8
0
500
1000
1500
Transcript Position
